# Dominant T-cell Receptor Delta Rearrangements in B-cell Precursor Acute Lymphoblastic Leukemia: Leukemic Markers or Physiological γδ T Repertoire?

**DOI:** 10.1097/HS9.0000000000000948

**Published:** 2023-09-01

**Authors:** Miriam Kelm, Franziska Darzentas, Nikos Darzentas, Michaela Kotrova, Wiebke Wessels, Sonja Bendig, Claudia D. Baldus, Marcus Lettau, Nicola Gökbuget, Dieter Kabelitz, Monika Brüggemann, Guranda Chitadze

**Affiliations:** 1 Medical Department II, Hematology and Oncology, Christian-Albrechts University of Kiel and University Hospital Schleswig-Holstein, Germany; 2 University Cancer Center Schleswig-Holstein, University Hospital Schleswig-Holstein, Kiel and Lübeck, Germany; 3 Institute of Immunology, Christian-Albrechts University of Kiel and University Hospital Schleswig-Holstein, Germany; 4 Department of Medicine II, Goethe University Hospital, Frankfurt, Germany

Acute lymphoblastic leukemia (ALL) is an aggressive hematological malignancy characterized by clonal expansion of aberrant B- and T-cell precursor lymphoid cells. Rearranged immunoglobulin or T-cell receptor (IG/TR) genes represent a DNA fingerprint of each lymphoid clone and are reliable markers for monitoring physiological and leukemic lymphocyte populations.^1^ Minimal or measurable residual disease (MRD) is the most important prognostic parameter predicting ALL relapse and is used for risk stratification and therapy adjustment.^2^ Therefore, accurate identification of leukemic IG/TR rearrangements for monitoring MRD is of high clinical importance. However, the reliable distinction of leukemia-associated IG/TR rearrangements from other clonally expanded lymphocyte populations is often complex and requires careful consideration, particularly due to the possibility of oligoclonality and the presence of cross-lineage IG/TR rearrangements in precursor leukemias. Both phenomena are presumably a consequence of aberrant, persisting recombination processes in malignant immature lymphoid cells.^3^ Depending on patients age and ALL subtype, a considerable number of B-cell precursor ALL (BCP-ALL) harbor TR cross-lineage rearrangements.^4^ For instance, TR delta (*TRD*) gene rearrangements occur in 40%–90% of BCP-ALL as cross-lineage rearrangements, especially in ALL cells arrested in early stages of differentiation, and are mostly restricted to incomplete TRDV2-TRDD3, TRDD2-TRDD3 rearrangements, or to combined TRDV-TRAJ recombinations, which are rare in normal lymphoid cells.^5–8^ Interestingly, we identified expanded complete TRDV-TRDJ clonotypes in diagnostic BCP-ALL samples, although they are not supposed to be rearranged in BCP-ALL. It is unclear if these rearrangements originate from leukemic cells or represent clonally expanded benign T-cell populations, most probably belonging to the γδ T-cell fraction, a minor subset of T cells with potent antileukemic activity,^9^ as αβ T cells regularly delete their TRD loci in the process of the formation of TRA rearrangements.^3^

To address this question, we have systematically analyzed TRD rearrangement profiles in diagnostic samples of 839 BCP-ALL patients (Figure 1A; 645 bone marrow aspirates, 193 blood samples, and 1 ascites) enrolled within the German Multicenter Adult Acute Lymphoblastic Leukemia (GMALL) Registry or the GMALL 08/2013 trial between 02/2016 and 05/2020. All patients gave informed consent to using residual material for research purposes. All diagnostic samples were analyzed using the amplicon-based next-generation sequencing Euroclonality-NGS TRD protocol, allowing amplification of all complete and incomplete rearrangements of the TRD locus, and combined TRD-TRAJ29 rearrangements in a single tube.^8^ Sequences were analyzed using ARResT/Interrogate pipeline.^10^

**Figure 1.**
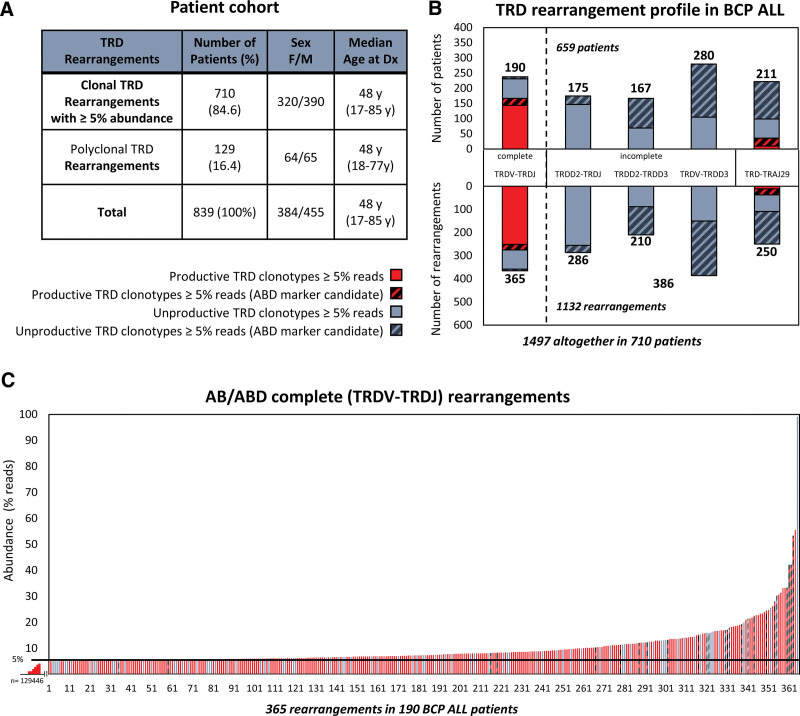
**TRD rearrangement profiles in a cohort of patients with BCP-ALL.** (A) Description of a cohort of 839 BCP-ALL patients with or without clonal TRD (≥5% reads) rearrangements. (B) Patients (upper panel) and their clonal TRD rearrangements (lower panel) identified using AB (filled) and ABD (dashed) are grouped based on junction class and TRD rearrangement profile. ABD TRD rearrangements were defined among AB TRD rearrangements. (C) Abundances (in % reads, y-axis) of all 365 marker-candidate complete TRDV-TRDJ rearrangements (x-axis). Red and gray bars represent productive and unproductive TRDV-TRDJ rearrangements, respectively. TRD clonotype read counts were converted in to a TRD clonotype cell counts in 305 of 365 complete TRDV-TRDJ rearrangements (154/190 patients) (Suppl. Table S1). AB = abundance-based approach; ABD = abundance and distribution-based approach; BCP-ALL = B-cell precursor acute lymphoblastic leukemia; MRD = minimal or measurable residual disease; *TRD* = TR delta.

We explicitly searched for clonal TRD rearrangements with an abundance of ≥5% reads (abundance-based [AB] marker screening, AB TRD), according to the cutoffs of the conventional MRD marker identification algorithm.^8^ The vast majority of BCP-ALL patients (84.6%, 710/839) harbored at least 1 marker-candidate clonal AB TRD rearrangement (Figure 1A). In total, 1497 clonal AB TRD rearrangements were identified at a median of 2, ranging from 0 to 9 rearrangements per patient. As expected, most of these AB TRD clones were incomplete TRDD2-TRDJ, TRDV-TRDD3, TRDD2-TRDD3 rearrangements or combined TRD-TRAJ29 junctions (Figure 1B; 75.6%, 1132/1497), in line with previous results of TRD rearrangement profiles of BCP-ALL.^5,11^ However, in 190 of 839 (22.6%) patients, we identified 365 complete TRDV-TRDJ rearrangements (at the median of 2, ranging from 1 to 7 rearrangements/patient), which are generally assumed not to represent BCP-ALL (Figure 1B, C; Suppl. Table S1).^5^ To increase the specificity, we next employed AB and distribution-based (ABD) marker screening approach on TRD rearrangements already predefined by AB approach, an ARResT/Interrogate tool allowing improved MRD marker identification. With the ABD approach, rearrangements are assessed as marker-candidates if they stand out from the background repertoire, significantly above their expected abundance according to the clonal distribution (Suppl. Figure S1). The ABD approach identified 40% of AB TRD marker-candidate rearrangements (587/1497), predominantly representing incomplete or TRD-TRAJ rearrangements (Figure 1B, dashed bars). Strikingly, the ABD algorithm also selected 31 complete TRDV-TRDJ rearrangements in 3.3% (28/839) of BCP-ALL patients (first column in Figure 1B, C). Of note, most of these complete TRDV-TRDJ rearrangements were productive (red bars), with 75.3% (275/365) and 77.4% (24/31) rearrangements in 19.9% (167/839) and 2.7% (23/839) BCP-ALL patients, when identified by AB (filled red) or ABD (dashed red) approach, respectively (Figure 1B, C). Comparable results were obtained from bone marrow and peripheral blood sample (Suppl. Figure S2).

We hypothesized that these complete TRDV-TRDJ rearrangements most likely represent accompanying γδ T-cell clones and not leukemia-derived cross-lineage TRD rearrangements.

We attempted to correlate the *TRD* gene rearrangement pattern with the suspected normal γδ T-cell subsets, which are typically defined by the variable fragment of the TRD chain. To this end, we studied the *TRDV* and *TRDJ* gene usage of identified complete TRDV-TRDJ rearrangements in the following patient groups with^1^ marker-candidate AB TRD rearrangements (blue bars),^2^ marker-candidate ABD TRD rearrangements (red bars) and compared it with^3^ all other TRDV-TRDJ rearrangements (<5%), potentially representing a polyclonal pool of γδ T cells (gray bars) (Suppl. Figure S3). Because only productive TRDV-TRDJ rearrangements form functional T-cell receptors and unproductive TRDV-TRDJ rearrangements rather represent the nonfunctional second allele in a given γδ T-cell clone,^12^ we primarily focused on productive TRD rearrangements. Detected frequencies indicated the dominant usage of *TRDV2* and *TRDJ1* gene segments (Suppl. Figure S3; upper panel for productive TRD rearrangements) in all 3 data sets, sequences corresponding to Vδ2+ γδ T cells, the most abundant circulating γδ T-cell population in adults (up to 90% of blood γδ T cells) and known to rearrange TRDV2-TRDJ1 preferentially.^13^

To further characterize the cell-of-origin of marker-candidate complete TRD rearrangements, we selected 13 patients with ABD TRD clones and tracked their kinetics during follow-up using highly sensitive digital droplet PCR, and compared their abundance to leukemia kinetics being assessed using routine IG/TR MRD analysis. Interestingly, ABD TRDV-TRDJ clones persisted at a relatively stable level (red lines) in discordance to ALL MRD dynamics (blue lines) in 12 of 13 patients (Figure 2A, left panel; Suppl. Table S2). In 1 of 13 patients, the ABD TRD rearrangement eventually became undetectable, similar to a malignant clone, however, with delayed kinetic (Figure 2A; right panel). Based on the availability of cryopreserved viable cells, we enriched γδ T cells using magnetic-activated cell sorting in 6 follow-up samples and quantified the abundance of the TRD marker before and after γδ T-cell enrichment using digital droplet PCR in 4 follow-up samples (Suppl. Material). Identified TRDV-TRDJ clones were 25-times (range 9–56, Figure 2B) more abundant following γδ T-cell enrichment, confirming that these rearrangements indeed mark persisting γδ T-cell populations, even if they can be rated as potential IG/TR MRD markers according to the commonly applied AB or intensified ABD marker identification thresholds. Other 2 samples were subjected to flow cytometric analysis for purity check due to insufficient material for paired analysis, showing γδ T-cell purity >90%. Interestingly, γδ T-cell proportions were relatively high in both samples (17% and 27% among T cells; Suppl. Table S3) before enrichment compared with generally expected values anywhere between 1% and 10% among T cells.^13^

**Figure 2.**
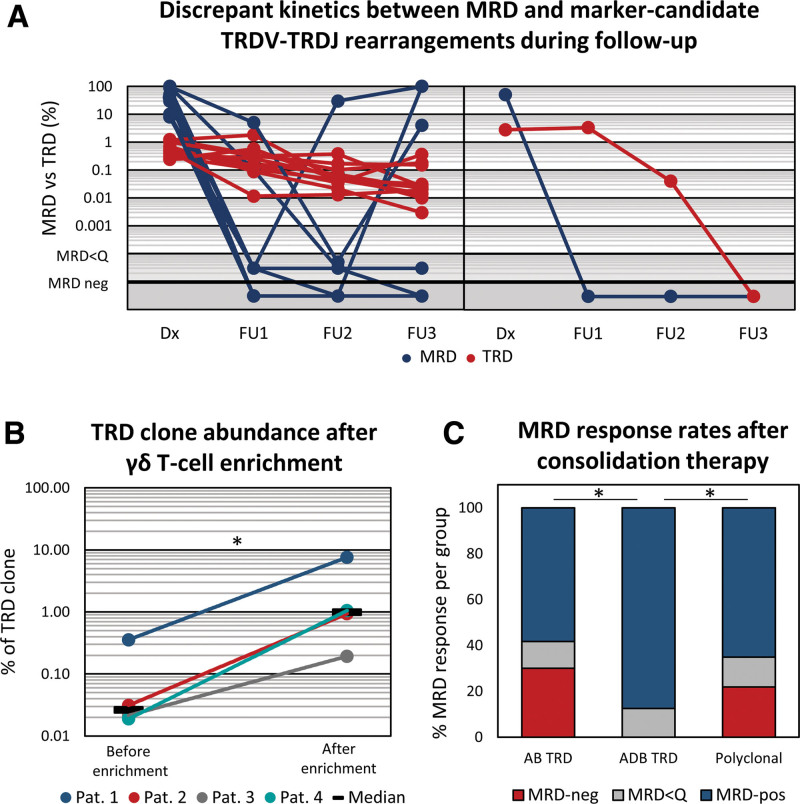
**Discrepant kinetics between MRD and marker-candidate complete TRDV-TRDJ rearrangements during follow-up.** Leukemia-defining IG/TR MRD kinetics is depicted (blue lines) together with abundance and distribution-based (ABD) marker-candidate complete TRDV-TRDJ rearrangements (red lines) at diagnosis and 3 follow-up time points of analyzed 13 patients with (A) persistent (n = 12) and (B) nonpersistent (n = 1) kinetics. Samples were selected based on the availability of longitudinal DNA samples among patients with ABD TRDV-TRDJ rearrangements. TRDV-TRDJ rearrangements were quantified using digital droplet PCR (Suppl. Table S4) (B) Abundance of TRDV-TRDJ clones before and after enrichment of γδ T cells in 4 follow-up samples. (C) MRD-response rates after consolidation chemotherapy from patients with productive TRDV-TRDJ AB TRD clones, ABD TRD, and without clonal AB TRD marker. Wilcoxon rank-sum test was performed to compare abundance of marker-like *TRD* clones. MRD-response rates between groups were compared using Fisher exact test or χ^2^ test. *indicates significant *p* value <0.05. AB = abundance-based approach; ABD = abundance and distribution-based approach; IG/TR = immunoglobulin or T-cell receptor; MRD = minimal or measurable residual disease; *TRD* = TR delta.

Altogether, these data strongly support the hypothesis that AB TRDV-TRDJ clonotypes represent physiological and possibly expanded γδ T-cell subpopulations, especially when being confirmed by ABD algorithm. Even if the role of these γδ T cells is unclear in this setting, they may contribute to cancer immune surveillance and improved leukemia control. Although representing a minor T-cell subset, γδ T cells are key sensors of cellular stress associated with cancer or infection and efficiently kill leukemic cells without the involvement of HLA molecules.^9,13,14^ Therefore, we categorized patients’ MRD response after induction and/or consolidation I either as MRD negative for patients in molecular remission, as MRD positive for patients with persistence of leukemia below the quantitative range (MRD<Q) or with quantifiable MRD positivity with a certain MRD level, according to the standard IG/TR MRD assessment criteria.^15^ Obtained MRD-response rates were compared between previously defined MRD-response groups with AB (1) and ABD (2) clonal TRDV-TRDJ rearrangements and patients without dominant TRDV-TRDJ rearrangements (3). Interestingly, the molecular remission rate after consolidation therapy was significantly (*p =* 0.013) higher in patients with dominant ABD TRDV-TRDJ rearrangements (87.5%, 12/14) compared with the remaining patients with 58.3% (70/120) and 65.1% (289/444) for AB clonal and polyclonal TRDV-TRDJ repertoire, respectively (Figure 2C). There was no significant difference among MRD-response rates at the other 2 time points. However, the proportions of patients with MRD positivity were consistently lower in patients with clonal ABD TRDV-TRDJ rearrangements at both time points (Red bars, Suppl. Figure S4). These results suggest that ABD TRDV-TRDJ clones represent expanded γδ T-cell population, possibly contributing to ALL immune surveillance.

In conclusion, clonal TRDV-TRDJ clonotypes in BCP-ALL most probably define accompanying γδ T-cell populations that persist over time and should not be used as MRD markers. Furthermore, our data suggest that high frequencies of ALL infiltrating bystander γδ T cells might contribute to ALL immune surveillance, leading to improved treatment outcomes. Finally, these findings warrant mechanistic studies in larger patient cohorts dissecting antileukemic functions of γδ T cells to explore novel strategies that maximize their cytotoxic potential in vivo or ex vivo as adoptive treatment strategies^13^ ultimately leading to a cure.

## ACKNOWLEDGMENTS

We thank Jennifer Sörensen for her expert technical assistance. Furthermore, the technical help of Petra Chall and Sandra Ussat is highly appreciated.

## AUTHOR CONTRIBUTIONS

MKe and FD collected patient data, performed experiments, and wrote first draft of the article. ND contributed to data analysis and interpretation of the results together with MKo, ML, and DK. SB performed flow cytometric measurements. NG provided patient information. GC and MB designed the research study, interpreted the results, and approved the final version of the article.

## DISCLOSURES

MB is contracted to carry out research for Affimed, Amgen, Regeneron, the advisory board of Amgen, Incyte, Speaker bureau of Amgen, Janssen, Pfizer, Roche. DK is a member of the Scientific Advisory Boards of Imcheck Therapeutics, In8Bio, Lava Therapeutics, and Phosphogam. NG is a member of the HemaSphere Editorial Board. All the other authors have no conflicts of interest to disclose.

## SOURCES OF FUNDING

This study was in part funded by the Deutsche Forschungsgemeinschaft (DFG, German Research Foundation)—project number 444949889 (KFO 5010/1 Clinical Research Unit’ CATCH ALL’ to GC, SB, CBD, and MB), through the “Clinician Scientist Program in Evolutionary Medicine” (project number 413490537 to GC). Deutsche José Carreras Leukämie-Stiftung (DJCLS, German Jose Carreras leukemia foundation), Grant/Award Number: DJCLS 22R/2019 and Deutsche Krebshilfe (project number 70113252). We acknowledge financial support by Land Schleswig-Holstein within the funding programme Open Access Publikationsfonds.

## Supplementary Material


