# Supplementary material for: Dominant T-cell Receptor Delta Rearrangements in B-cell Precursor Acute Lymphoblastic Leukemia: Leukemic Markers or Physiological γδ T Repertoire?
Source: Hemasphere. 2023 Sep 1;7(9):e948. doi: 10.1097/HS9.0000000000000948 (PMC10476800; doi:10.1097/HS9.0000000000000948)
Supplement: Supplementary file 2 [file hs9-7-e948-s002.pdf]

Suppl. Figure 1

Abundance and distribution-based (ADB) marker screening approach

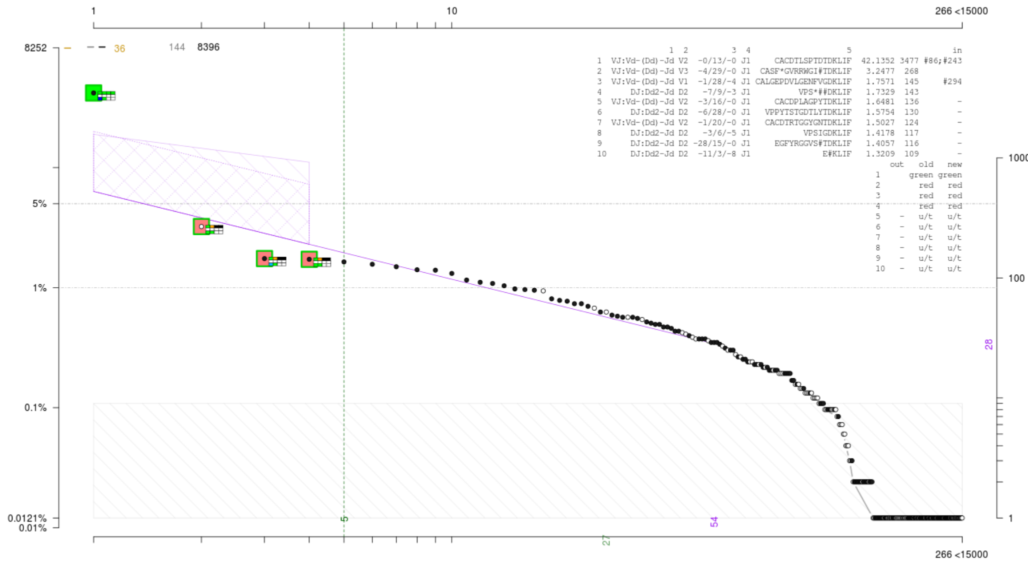

Suppl. Figure 1: Visualisation of the algorithmic abundance and distribution-based marker screening approach (ADB) created by ARResT/Interrogate. Each clonotype detected in the TRD library is represented as a dot on an abundance (y-axis) to rank (x-axis) double logarithmic scale. The most abundant rearrangements are evaluated as potential markers based on the following criteria: sample diversity (inverse Simpson index), read abundance and the distance to the expected abundance based on the sample distribution (violet line). The box-colors rate if the rearrangement is likely to be a marker. In this example the clonotypes with red boxes were unlikely to be leukemic markers, while the top clonotype with the green box, showing a read abundance above 5%, which is also exceeding the expected abundance based on the sample distribution (violet line) was consequently accepted as an eligible MRD marker.

Suppl. Figure 2

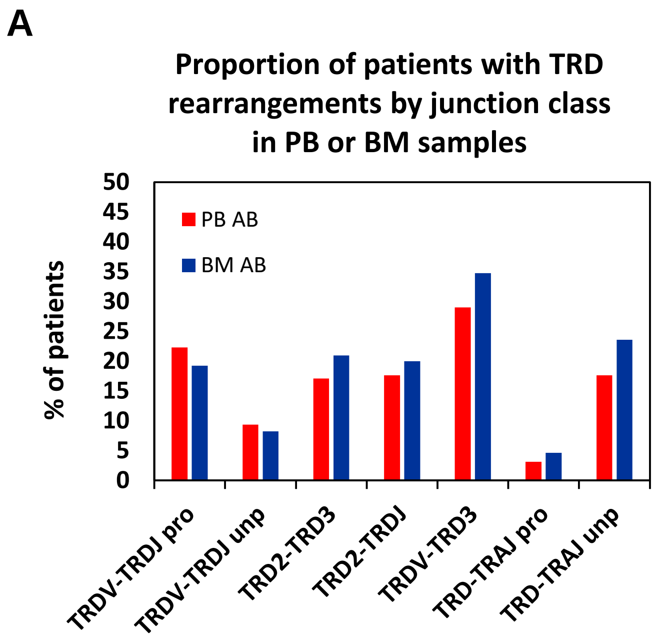

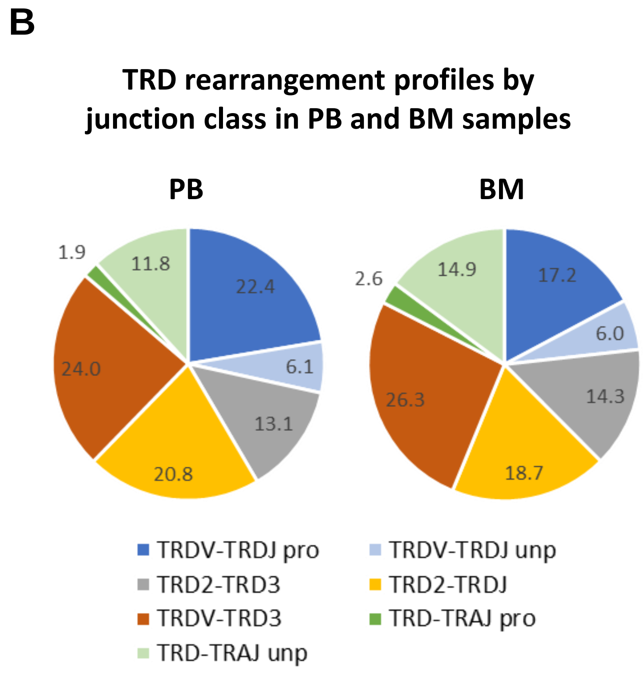

C

Complete TRDV-TRDJ rearrangements with ≥ 5% abundance in PB and BM samples

	PB	BM	PB	BM
	AB TRDV-TRDJ		ABD TRDV-TRDJ	
Average number of clones / per patient	1.8	2	1	1.2
Number of TRDV-TRDJ clones (Number of patients)	89 (50 pts)	279 (139 pts)	12 (12 pts)	19 (16 pts)
Median number of detected TRDV-TRDJ clones (range)	1 (1-4)	1 (1-5)	1 (1-1)	1 (1-2)
Median abundance (%) of TRDV-TRDJ clones (range)	7 (5 - 42.2)	7.4 (5-99)	18.2 (5.1-42.2)	15.3 (5.6-53.3)

Suppl. Figure 2: Sample origin does not influence the frequency or the profile of TRD rearrangements . (A) Proportion of patients harboring at least one TRD rearangement in any of depicted junction class does not differ among 193 peripheral blood (PB) and 645 bone marrow (BM) samples. (B) Abundances of each junction class TRD rearrangements are comparable between PB (313 rearrangements) and BM (1183 rearrangements). (C) Average number, median clone number and the median abundance (% of reads) of complete abundance-base (AB) and abundance and distribution-based (ABD) TRDJ-TRDV rearrangements are shown in PB and BM samples.

Suppl. Figure 3

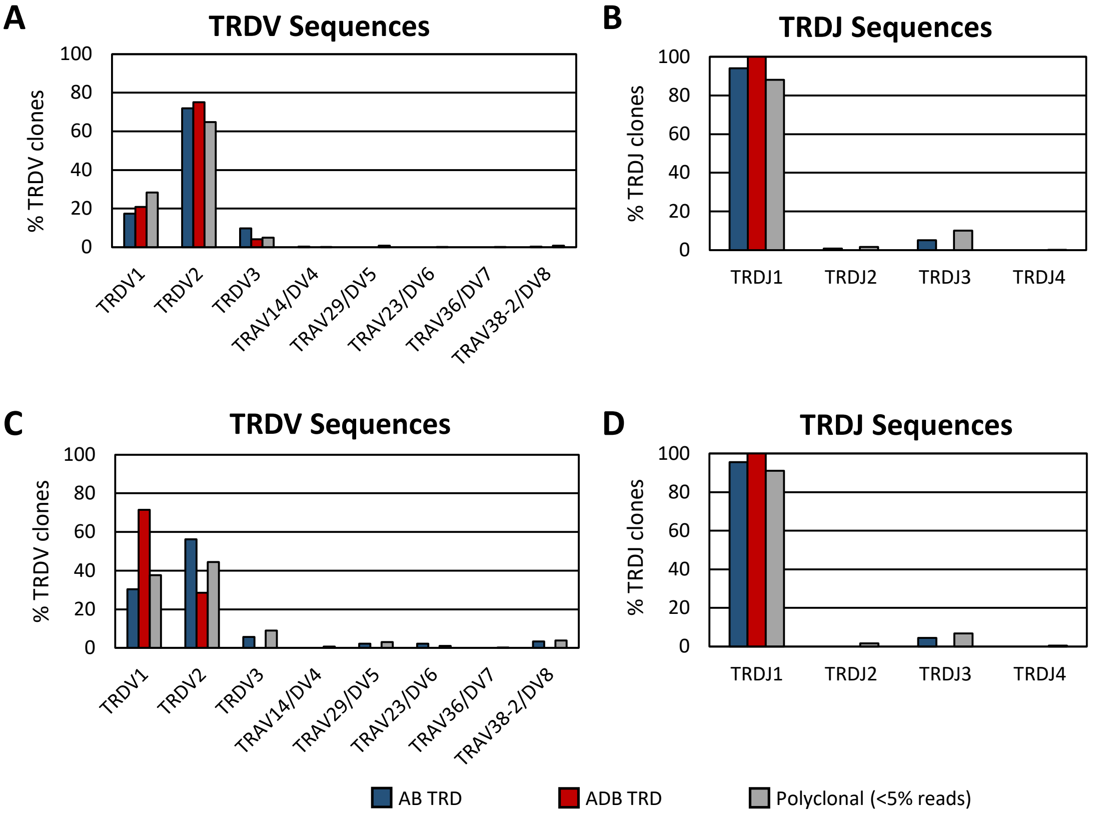

Suppl. Figure 3: TRDV- and TRDJ usage of productive and unproductive TRDV-TRDJ clonotypes among clonal AB TRD (blue), ADB TRD (red) and polyclonal TRDV-TRDJ rearrangements (<5% reads) (grey) A. V usage of productive TRD clones. B. J usage of productive TRD clones. C. V usage of unproductive TRD clones. D. J usage of unproductive TRD clones.

Suppl. Figure 4

MRD response rates in BCP ALL patients with TRDV-TRDJ rearrangements

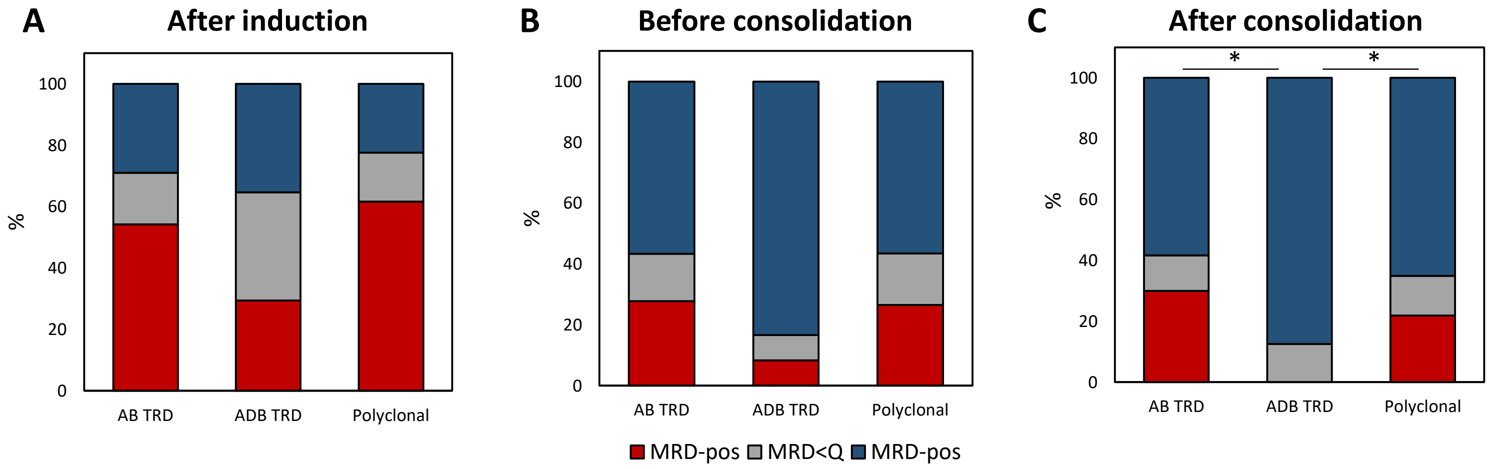

After Ind I	MRD-pos patients (%)	MRD<Q Patients (%)	MRD-neg Patients (%)
AB TRD	71 (54,2 %)	22 (16,8 %)	38 (29,0 %)
ADB TRD	5 (29,4 %)	6 (35,3 %)	6 (35,3 %)
Polyclonal	332 (61,6 %)	86 (15,9 %)	121 (22,5 %)
Total	403	108	159

Before Cons I	MRD-pos patients (%)	MRD<Q Patients (%)	MRD-neg Patients (%)
AB TRD	25 (27,8 %)	14 (15,6 %)	51 (56,7 %)
ADB TRD	1 (8,3 %)	1 (8,3 %)	10 (83,3 %)
Polyclonal	103 (26,5 %)	66 (17,0 %)	220 (56,5 %)
Total	128	80	271

After Cons I	MRD-pos patients (%)	MRD<Q Patients (%)	MRD-neg Patients (%)
AB TRD	36 (30,0%)	14 (11,7 %)	70 (58,3 %)
ADB TRD	0 (0,0%)	2 (12,5 %)	14 (87,5 %)
Polyclonal	97 (21,8 %)	58 (13,1 %)	289 (65,1 %)
Total	133	72	359

Suppl. Figure 4: MRD Response in BCP ALL patients grouped based on their TRD rearrangement profiles. Comparison of MRD response rates in BCP ALL patients with only productive TRDV-TRDJ clones identified with abundance-based (AB TRD) and abundance and distribution-based (ABD TRD) approach and in patients with polyclonal TRDV-TRDJ following Induction I (A, 670 patients), before Consolidation I (B, 479 patients) and after consolidation I (C, 564 patients).
