# Supplementary material for: Dominant T-cell Receptor Delta Rearrangements in B-cell Precursor Acute Lymphoblastic Leukemia: Leukemic Markers or Physiological γδ T Repertoire?
Source: Hemasphere. 2023 Sep 1;7(9):e948. doi: 10.1097/HS9.0000000000000948 (PMC10476800; doi:10.1097/HS9.0000000000000948)
Supplement: Supplementary file 4 [file hs9-7-e948-s004.docx]

**Supplemental Data**

**Amplicon-based Next-generation sequencing (NGS)**

For amplicon-based NGS marker detection, the use of the EuroClonality-NGS TRD primer set on 100ng of 839 diagnostic DNA (645 bone marrow aspirates, 193 blood samples, 1 ascites) allowed the amplification of complete TRDV-TRDJ and incomplete TRDD2-TRDJ, TRDD2-TRDD3, TRDV-TRDD3, as well as TRD-TRAJ29 rearrangements. Samples were sequenced on a MiSeq (Illumina, San Diego, CA, USA) with 2×250bp reads (1). Sequences were analyzed with ARResT/Interrogate (freely available online at arrest.tools/interrogate) (2). Identification of marker rearrangements, was performed according to conventional MRD marker screening recommendations, based on an abundance threshold (≥ 5% usable reads) in the first instance (‘abundance-based’ marker screening (AB)). In order to improve the likelihood of identifying real monoclonal leukemic rearrangements instead of overamplified background clones, we further applied an ‘abundance and distribution-based’ approach (ADB), which among other criteria, assesses whether rearrangements stand out from the rest of the sample, significantly above their expected abundance according to the Patero distribution (for details see Suppl. Figure 1) (3). In the presence a standardized human cell line-based DNA reference sample, which is spiked into a patient DNA sample (central in-tube QC, cIT-QC), clonotype read counts were converted into cell counts, enabling indirect assessment of proportion of the cells harboring rearrangements of interest (Suppl. Table 1) (4). This approach is available on <http://arrest.tools/interrogate> under the Interrogate.Marksman user mode. TRD rearrangement profiles were compared also between bone marrow (n = 645) and peripheral blood samples (n=193) (Suppl. Figure 2).

**Magnetic activated cell sorting (MACS)**

Frozen PBMCs of 6 patient samples were thawed in 37 °C RPMI 1640 medium with 10% FCS and washed twice with 5 ml PBS before performing the MACS. The *TCRγ/δ^+^ T Cell Isolation Kit, human* in combination with LS Columns and MACS Separator (Miltenyi Biotech, Bergisch Gladbach, Germany) was used for manual enrichment of γδ T cells following manufacturer`s protocol. The enrichment efficacy was assessed via flow cytometry in two patient samples and enriched material from other four samples was subjected to DNA extraction with guanidinium isothiocyanate (GITC).

**Flow cytometry**

The enrichment of γδ T cells throughout the MACS was evaluated by flow cytometry. 150 000 mononuclear cells of initial sample (prior enrichment) and all available cells after enrichment step (ca. 20 000 cells remaining) were stained with anti-CD3-BV786 (SK7, BD, Franklin Lakes, NJ, USA), anti-CD45-AF700 (2D1, Biolegend, San Diego, CA, USA), anti-CD4-APC-H7 (RPA-T4, BD, Franklin Lakes, NJ, USA), anti-CD8-BV510 (SK1, BD Franklin Lakes, NJ, USA), anti-TCRγδ-PECy7 (11F2, BD, Franklin Lakes, NJ, USA), anti-TCR-Vd1-FITC (TS8.2, Invitrogen, Waltham, MA, USA) and anti-TCR-Vd2-BV711 (B6, Biolegend San Diego, CA, USA) monoclonal antibodies for 20 minutes followed by two washing FACS washing buffer containing 1% BSA in PBS. Samples were measured to assess γδ T-cell purity (Suppl. Table 3) on BD FACS Lyric TM Flow Cytometer (BD, Franklin Lakes, NJ, USA) and analyzed using FlowJo™ Software version 10.8.1 (BD Bioscience, San Jose, CA, USA).

**DNA-Extraction with guanidinium isothiocyanate (GITC)**

A GITC and silica gel-based method was used for the DNA extraction of isolated cells(5). The DNA concentration was measured with a Nanodrop ND-1000 (NanoDrop Technologies, Inc., Wilmington, DE, USA) and the amplifiability was checked via albumin real time PCR.

**Albumin real time PCR**

To determine the albumin copies of the extracted DNA, one microliter was used for albumin real time PCR using albumin specific forward and reverse primers as well as an albumin specific fluorescent dye. The PCR was performed on a LightCycler® 480 Real-Time PCR System (Roche Dignostics, Rotkreuz, Switzerland) at 58 °C. The albumin copies were calculated with a serial dilution with known concentrations that was performed on the same PCR run.

**Digital droplet PCR (ddPCR)**

The QX200 Droplet Digital PCR System (Bio-Rad laboratories, Hercules, CA, USA) was used in combination with patient-specific reverse primers, TRDV1, TRDV2 or TRDV3 specific forward primers and a 6-FAM-phosphoramitid dye to track the kinetics of the detected γδ clones over four time points and the MACS enriched clones (Suppl. Table 4). Triplicates of every time point were performed to achieve a sensitivity of 3.2x10^-5^. The data was analyzed with QuantaLife Software. Only replicates with ≥ 9000 droplets were accepted and the threshold was adjusted as close to the background signal as possible. The calculated copies of the target per well were normalized to the corresponding albumin quantities, which were analyzed routinely after DNA extraction via real-time PCR to determine the abundance of the target gene.

**Statistical methods**

MRD response rates between groups were compared using Fisher exact test or Chi-square test and Wilcoxon rank-sum test was performed for continuous variables.

**References**

1. Bruggemann M, Kotrova M, Knecht H, Bartram J, Boudjogrha M, Bystry V, et al. Standardized next-generation sequencing of immunoglobulin and T-cell receptor gene recombinations for MRD marker identification in acute lymphoblastic leukaemia; a EuroClonality-NGS validation study. Leukemia. 2019;33(9):2241-53.

2. Bystry V, Reigl T, Krejci A, Demko M, Hanakova B, Grioni A, et al. ARResT/Interrogate: an interactive immunoprofiler for IG/TR NGS data. Bioinformatics. 2017;33(3):435-7.

3. Koch H, Starenki D, Cooper SJ, Myers RM, Li Q. powerTCR: A model-based approach to comparative analysis of the clone size distribution of the T cell receptor repertoire. PLoS Comput Biol. 2018;14(11):e1006571.

4. Knecht H, Reigl T, Kotrova M, Appelt F, Stewart P, Bystry V, et al. Quality control and quantification in IG/TR next-generation sequencing marker identification: protocols and bioinformatic functionalities by EuroClonality-NGS. Leukemia. 2019;33(9):2254-65.

5. Extraction of DNA from the whole blood by silica gel. [Internet]. 1995. Available from: <http://www.protocol-online.org/cgi-bin/prot/view_cache.cgi?ID=1161>.
